# Utility of the Pleth Variability Index in predicting anesthesia-induced hypotension in geriatric patients

**DOI:** 10.3906/sag-1912-132

**Published:** 2021-02-26

**Authors:** Ahmet YÜKSEK

**Affiliations:** 1 Department of Anesthesiology and Reanimation, Bozok University, Yozgat Turkey

**Keywords:** Pleth Variability Index, geriatric assessment, hypotension

## Abstract

**Background/aim:**

Anesthesia-induced hypotension may have negative consequences in geriatric patients. Therefore, predicting hypotension remains an important topic for anesthesiologists. Pleth Variability Index (PVI) measurement provides information about the fluid status and vascular tonus of patients. In this study, the ability of the Pleth Variability Index to predict hypotension after general anesthesia induction was evaluated.

**Materials and methods:**

PVI values ​​obtained from pulse oximetry were recorded, in addition to preoperative standard anesthesia monitoring. The correlation between the PVI value and mean arterial pressure (MAP), systolic arterial blood pressure (SAP) changes, and the power of PVI values ​​to predict the incidence of hypotension after anesthesia induction (>20% MAP decrease) was tested.

**Results:**

Eighty patients over 65 years of age who were operated under general anesthesia were included in the study. Hypotension was observed in 20 patients (25%). PVI values were mild and positively correlated with MAP changes (r = 0.195 and P = 0.041). According to receiver operating characteristic (ROC) analysis, the incidence of hypotension increased in patients with PVI values above 15.45%. We also found the following diagnostic results for PVI value for predicting hypotension: P = 0.044 and area under the ROC curve of 0.651 ± 0.073 (95% confidence interval (CI): 0.507–0.794), 40% sensitivity, 80% specificity, a PPV of 40%, an NPV of 80%, a cut-off value of 15.45, a positive likelihood ratio of 2, a negative likelihood ratio of 0.75, and a Youden Index of 0.2.

**Conclusion:**

Predicting hypotension in geriatric patients is an important issue for anesthesiologists. As an easily applicable test, the Pleth Variability Index is useful in predicting MAP reduction in patients. This practical technique can be used routinely in all geriatric patient groups.

## 1. Introduction

The average age in developed countries is increasing; as such, geriatric anesthesia is an emerging field of study that aims to better serve this special patient population [1]. General anesthesia in geriatric patients is a high-risk procedure due to the effects of drugs, mechanical ventilation, and possible comorbidities [2].

Hypotension due to the cardiodepressant and vasodilator effects of anesthetic drugs in the induction of general anesthesia may cause decreased critical organ perfusion and increase cardiac and cerebral complications [3]. The risk for complications increases, even with short-term hypotension [4]. Moreover, because laparoscopic surgeries have a suppressive effect on the respiratory and circulatory systems, it is important to be able to predict the development of hypotension in these patients before surgery [2]. Increased fluid shortage in patients due to prolonged waiting times for operations may also lead to the development of hypotension.

The peripheral perfusion index (PI) reflects the amplitude of the pulse oximeter waveform and is calculated as the pulsatile infrared signal (variable component), indexed against the nonpulsatile infrared signal (constant component). PI is expressed as a percentage (0.02%–20%). The Pleth Variability Index (PVI) is a measure of the dynamic changes in the PI that occur during one or more complete respiratory cycles. PVI may show changes that reflect physiologic factors such as vascular tone, circulating blood volume, and intrathoracic pressure excursions [5]. In this way, PVI provides information about the fluid status of patients by decreasing or increasing peripheral perfusion according to changes in respiration [6]. The ratio of pulsatile to nonpulsatile pulse oximetry measurements corresponds to PI, and the ratio of the highest and lowest PI values ​​corresponds to the PVI; there is no defined reference range [7,8].

Tsuchiya et al. demonstrated that the PVI could be used to evaluate anesthesia-induced hypotension in patients undergoing general anesthesia without age group classification [8]. Similar studies have been conducted in pregnant women undergoing spinal anesthesia and, again, PVI was found to be a successful tool for predicting hypotension [9,10]. This technique has been used in patients undergoing mechanical ventilation in the intensive care unit to detect fluid responsiveness through changes in respiratory patterns and peripheral perfusion [11]. It is a relatively new area of interest in the use of spontaneous breathing before anesthesia.

Predicting hypotension in geriatric patients with multiple comorbidities is valuable for early intervention and the reduction of complication rates. However, PVI and its predictive power in anesthesia-induced hypotension have not been extensively studied in geriatric patient populations. The aim of this study, therefore, was to test the utility of PVI in predicting anesthesia-induced hypotension in geriatric patients undergoing laparoscopic surgery. To the best of our knowledge, this is the first study on the use of PVI on anesthesia-induced hypotension in geriatric patients.

## 2. Materials and methods

Previous studies involving similar patient populations were examined, and power analyses revealed that at least 50 patients were required [9,12]. After approval from the local ethics committee (2017-KAEK-189_04), American Society of Anesthesiology (ASA) class 1 and class 2 patients, who underwent laparoscopic surgery at a university hospital and agreed to participate, were included in the study. The protocol adhered to the principles of the Declaration of Helsinki. This study was carried out prospectively and observationally between November 2018 and December 2019.

Patients with known cardiovascular diseases (all arrhythmias, including atrial fibrillation, uncontrolled hypertension, and congestive heart failure), those using vasoactive drugs (betablockers), and those with peripheral vascular diseases or neuropathy were excluded from the study. 

Noninvasive blood pressure, heart rate, pulse oximetry, and PVI values which were automatically calculated from pulse oximetry were measured in the operating room after the patients rested for 5 min. The preoperative PVI value taken at this point was recorded as basal PVI. Preoperative total fasting times were also recorded.

After standard monitoring, an intravenous balanced crystalloid solution was started at a standard rate of 100 mL/h and was adjusted according to patient condition after intubation. Induction of anesthesia was performed using 2-mg/kg propofol and 0.6-mg/kg rocuronium, in accordance with hospital standards. After induction, the patients were intubated after being ventilated with a mixture of 80% oxygen and 20% air for 3 min with a peak pressure below 30-cm H_2_O. Hypotension was defined as a decrease in mean arterial pressure (MAP) >20% after anesthesia induction and treated with intermittent bolus doses of 5-mg ephedrine. The values ​​in patients with hypotension were not recorded after ephedrine administration. The definition of hypotension was based on previous studies [13]. Noninvasive blood pressure measurements were performed at 30-s intervals during the first 3 min after induction. The lowest systolic arterial blood pressure (SAP) was recorded; oxygen saturation and heart rate values ​​were also recorded at this point. Apart from our study protocol, we did not perform a postoperative follow-up related to outcomes in hypotensive patients.

Patients were divided into 2 groups (hypotensive and normotensive), and the correlation between the preoperative anesthetic baseline PVI value and preoperative heart rate, preoperative SAP, preoperative MAP, SAP after induction, and changes in blood pressure values ​​were analyzed for these 2 groups.

SPSS version 22.0 (IBM Corporation, Armonk, NY, USA) was used for statistical analysis. The Kolmogorov–Smirnov normality test was performed to examine the distribution of the measured values. The correlation between PVI and preoperative and postinduction systolic arterial pressure values was investigated using Pearson’s correlation analysis. The predictive power of PVI for hypotension was evaluated by using receiver operating characteristic (ROC) curve analysis, and the independent samples t-test was used to analyze normally distributed quantitative data; the chi-square (c2) test was used to compare qualitative data. A value of P < 0.05 was accepted as statistically significant [14].

## 3. Results

Eighty patients over 65 years of age who underwent laparoscopic surgery were included in the present study. No patient refused to participate in the study after enlightenment. The mean age of the patients was 72.97 ± 5.20 years old (range: 65–87), and there was no difference in age, sex, height, weight, or ASA classification between the hypotensive and normotensive patients. The demographic data of the patients included in the study are summarized in Table 1. The mean fasting times of hypotensive and normotensive patients were 9.17 ± 2.43 and 9.80 ± 2.09 h, respectively. There was no significant difference in the fasting time between groups (P = 0.271)

**Table 1:** Demographic data.

Characteristics	Hypotensive (n = 20)	Normotensive (n = 60)	P-value
Age (years)	75.1 ± 6.17	72.26 ± 4.68	0.071
Sex (F/M), n/n	12/8	35/25	0.086
Height (cm)	162.21 ± 3.92	168.12 ± 4.4	0.066
Weight (kg)	72.0 ± 5.8	69.8 ± 6.4	0.073
ASA class I/II, n/n	10/10	34/26	0.080

Data presented as mean ± standard deviation unless otherwise indicated. ASA: American Society of Anesthesiologists.

Hypotension, which we accepted as more than a 20% reduction in MAP, was observed in 20 (25%) patients after anesthesia induction. The mean systolic arterial pressure change was 19.63% in all patients and 36.41% in hypotensive patients. 

Although there was a difference between hypotensive and normotensive patients in terms of PVI values (P = 0.022), there was no significant difference in MAP, SAP, and HR values. The difference between variables in hypotensive and normotensive patients is shown in Table 2.

**Table 2:** Variables in hypotensive and normotensive patients.

	Hypotensive	Normotensive	P-value
PVI	14.12 ± 4.51	11.68 ± 3.87	0.022
MAP	107.30 ± 10.48	89.20 ± 12.60	0.066
SAP	135.20 ± 12.42	119.35 ± 14.01	0.112
HR	74.15 ± 12.09	82.65 ± 13.73	0.241

PVI: Pleth Variability Index; MAP: Mean arterial pressure, (mmHg); SAP: Systolic arterial blood pressure (mmHg): HR: Heart rate, beats per minute (bpm).

The mean baseline PVI​​ of the patients before anesthesia was 12.29 ± 4.15. When the relationship between preoperative MAP, preoperative SAP, and preoperative heart rate was examined, both in hypotensive and normotensive patients, as expected, a positive and significant correlation was observed between preoperative MAP and SAP values (P < 0.001). Neither MAP nor SAP correlated with preoperative heart rate (P > 0.05). Table 3 shows the significant correlation of SAP and MAP values.

**Table 3:** Correlations among MAP_1, SAP_1, and HR_1.

	Measurement		MAP_1	SAP_1
Hypotensive	HR_1	r	–0.049	–0.098
		P	0.663	0.387
	SAP_1	r	0.910	1
		P	<0.001*	
Normotensive	HR_1	r	0.121	0.028
		P	0.287	0.080
	SAP_1	r	0.887	1
		P	<0.001	

*Pearson’s correlation test, significant at the 0.01 level. HR_1 = preoperative heart rate (bpm); SAP_1 and MAP_1 = preoperative systolic and mean arterial blood pressure (mmHg), respectively.

Pearson’s correlation test was used for correlation analysis between the basal PVI value and parameters that could be a risk factor in the development of hypotension. A positive and mild correlation was found between basal PVI values and MAP change (r = 0.195 and P = 0.041). There was no significant correlation between preoperative the MAP value and PVI (r = 0.137 and P = 0.062) between preoperative SAP and PVI (r = 0.164 and P = 0.146) and between the preoperative heart rate and PVI (r = 0.112 and P = 0.321). MAP, SAP, and PVI correlations are summarized in Table 4.

**Table 4:** MAP, SAP, and Pleth Variability Index correlations.

		Pleth variability index
MAP change	rP	0.1950.041*
Preoperative MAP	rP	0.1370.062
Preoperative SAP	rP	0.1640.146
Preoperative HR	rP	0.1120.321

*Correlation significant at the 0.05 level. HR = Heart rate (bpm); SAP and MAP = Systolic and mean arterial blood pressure (mmHg), respectively.

The ability of the basal PVI value to differentiate hypotension was examined using ROC analysis, and the mean area under the ROC curve (AUC) was 0.651 ± 0.073 (95% confidence interval (CI): 0.507–0.794) was found to be statistically significant in predicting hypotension (P = 0.04). The cut-off PVI value was calculated to be 15.45. The positive predictive value (PPV) was calculated as 40%, and the negative predictive (NPV) value was calculated as 80%. Hypotension was observed in 42% of patients with a basal PVI >15.45 and 23% in patients with a basal perfusion index <15.45. There were 8 hypotensive and 12 normotensive patients with a PVI value above the threshold value and 12 hypotensive and 48 normotensive patients with a PVI value below the threshold value. Likelihood ratios in diagnostic testing were calculated as LR+: 2 and LR–: 0.75. Also, the Youden Index was calculated as 0.2. The power of basal PVI values ​​to predict the incidence of hypotension, according to ROC analysis, is shown in Figure.

**Figure:**
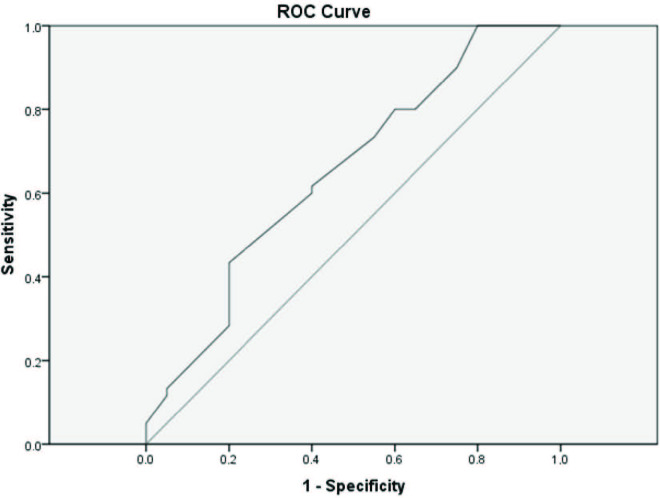
Receiver operating characteristic (ROC) curve analysis for Pleth Variability Index to predict the incidence of hypotension. Mean ± SD, area under the ROC curve: 0.651 ± 0.073 (95% CI: 507–0.794; P = 0.044); sensitivity of 40%, specificity of 80%, PPV of 40%, NPV of 80%, and cut-off value of 15.45.

When MAP, SAP, and HR values and their ability to predict hypotension development were analyzed by ROC analysis, the results were as follows: for MAP: AUC 0.738, standard deviation (SD) 0.002, CI: 95% (0.628–0.847) and P = 0.056; for SAP: AUC 0.659, SD: 0.034, CI: 95% (0.525–0.793) and P = 0.068; and for HR: AUC 0.311, SD: 0.012, CI: 95% (0.174–0.447) and P = 0.070. These parameters were not predictive.

## 4. Discussion

Geriatric patients require more surgical procedures than the general population. General anesthesia provides greater hemodynamic stability than spinal anesthesia; however, there is a risk for hypotension with vasodilator and the cardiodepressant effects of anesthetic drugs. Older age alone is a risk factor for the development of hypotension [14]. In this patient group, hypotension should be recognized early and treated quickly to reduce the complication rate.

PVI uses photoplethysmographic data obtained from pulse oximetry and perfusion index data, providing a ratio of pulsatile to nonpulsatile signals. Increased intrathoracic pressure with respiration will lead to more immediate reductions in peripheral perfusion in patients with a fluid deficit. In this case, a decrease in the PI value of the patient will be observed. As a result of these changes with respiration, the ratio of the highest and lowest PI ​​corresponds to the PVI. High PVI values are observed in patients with a high fluid deficit or in those who do not respond to fluid application with changes in the PI [11]. PVI was used in the regulation of perioperative fluid therapy in geriatric patients [15]. This was the first purpose of this technique. However, predicting hypotension in anesthesia induction and being able to do this specifically in a group of patients at high risk such as geriatric patients will contribute to our anesthesia practice. In this sense, we think that our study can also contribute to geriatric anesthesia.

According to the findings from our study, with preoperative baseline values, the PVI can predict the development of hypotension in geriatric patients undergoing laparoscopic surgery as a noninvasive and easily applicable test. Similar studies have been conducted for pregnant patients under spinal anesthesia or for those under general anesthesia without age limitations. These results are also consistent with our study [8,10,16].

In our study, the incidence of hypotension was increased in patients with PVI >15.45 before general anesthesia. This value was calculated to be 18 in the study by Kuwata et al. and 15 in Tsuchiya’s study [8,10]. Previously, PVI was used in studies investigating the relationship between respiratory movements and changes in the peripheral PI in patients undergoing mechanical ventilation. PVI has significant predictive power in terms of fluid deficit or nonresponse to fluid treatment. Since PVI is a ratio of PI values, there is no defined reference range. However, we think that the value of 15.45 in our study and the results in similar studies will make sense in clinical practice and that a new threshold can be drawn which can be defined as high or low Pleth variability with new data [8,10,16]. Hypotension was observed in 42% of patients with a basal PVI >15.45 and in 23% in patients with a basal perfusion index <15.45. Calculating positive and negative predictive values as 40% and 80% indicates that the predictive power is at medium levels. These values were 80% for PPV and 61.4% for NPV in Sun’s study. In Kuwata’s study, these values were calculated as 78% for PPV and 83% for NPV. However, these studies evaluated patients undergoing elective cesarean surgery. When our study results and predictive values are evaluated together, they support our idea of using PVI results as an early warning system instead of a sharp distinction.

Systemic diseases, especially vascular problems and hypertension, may affect the results obtained with PVI. The fact that these problems are also commonly seen in geriatric patients may limit the use of PVI. We think that it may be necessary to carry out new studies on the use of PVI in systemic diseases. Although some recent studies have indicated that preoperative or basal heart rate has a predictive value for hypotension and cardiac complications, there was no significant relationship between basal heart rate and hypotension development in our study groups (Table 3) [17–19].

 It is noteworthy that there was a correlation between PVI and MAP change (P = 0.042), but there was no significant relationship between PVI and preoperative MAP values. (r = 0.062) (Table 4). We believe that the relationship between these 2 variables should be supported by more studies. 

In our study, it was observed that this test was easily used in patients who were awake in the preoperative waiting room. Our study involving geriatric patients undergoing general anesthesia and previous studies in cesarean section patients undergoing spinal anesthesia conclude that the routine use of this test may be beneficial [16]. The positive features of this test include its noninvasiveness, its ease of performance, and the fact that it is inexpensive. However, there are insufficient data to distinguish the cause of hypotension due to peripheral vasodilatation and fluid redistribution or due to cardiac output decrease after general anesthesia. In this distinction, our patients were selected from nonheart disease participants to investigate the hypotension that may result from a fluid deficit or decreased vascular tone. However, the extent to which this highly selected patient group represents all geriatric patients is controversial, and this may be considered one limitation of our study. Polypharmacy and malnutrition, which are more common in geriatric patients, were not considered in the study. We think that this issue should also be mentioned as a limitation of our study. We think that it may be misleading to add multivariate analysis without examining all possible factors. It may even be an advantage to detect increased risk without having to identify possible causes of hypotension. Regardless of the risk factors, its use in finding patients with increased hypotensive risk supports the hypothesis.

## 5. Conclusion

The ability to predict hypotension in all patients, especially geriatrics, would be highly convenient for anesthetists and provide safety to patients. As an easily applicable test, PVI is useful in predicting the development of hypotension. It can be used as an early warning system. High PVI values ​​can be interpreted, and the incidence of MAP decrease will be higher after anesthesia induction in geriatric patients.

## Informed consent

Local ethics committee approval was received for this study (Bozok University Ethical Committee, 2017-KAEK-2019-189_04).
